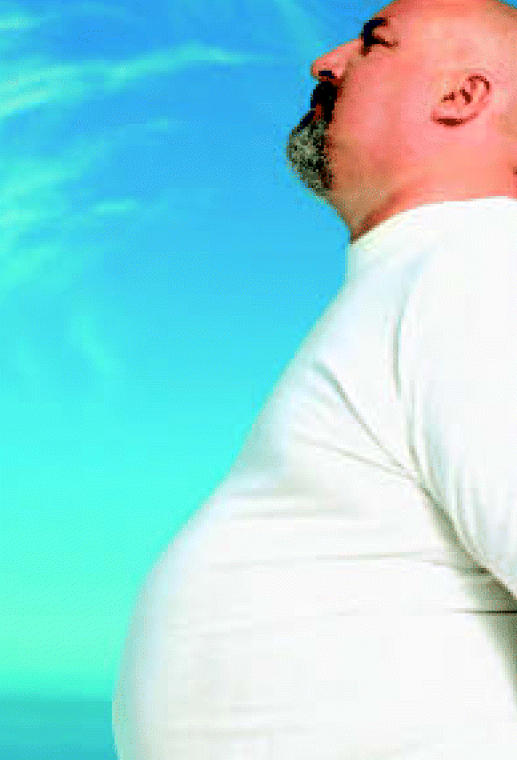# The Beat

**Published:** 2006-11

**Authors:** Erin E. Dooley

## Lower Prevalence of Breast Cancer Gene Mutations

Approximately 200,000 women are diagnosed with breast cancer each year. A paper in the 15 August 2006 issue of *Cancer Research* now gives the clearest picture to date of how many people in the United States carry mutations in the two dominant “breast cancer genes,” *BRCA1* and *BRCA2*. The authors wrote that 2.4% of the breast cancer patients in their study had *BRCA1* mutations, whereas 2.3% had *BRCA2* mutations. They also found that among white and black women aged 35 to 64 in the general population, the prevalence of *BRCA1* mutations is 0.06% and that of *BRCA2* mutations is 0.4%. The results are largely compatible with earlier estimates. Germline mutations in these genes are associated with a 26–84% lifetime risk of breast cancer and a 10–50% lifetime risk of ovarian cancer.

**Figure:**
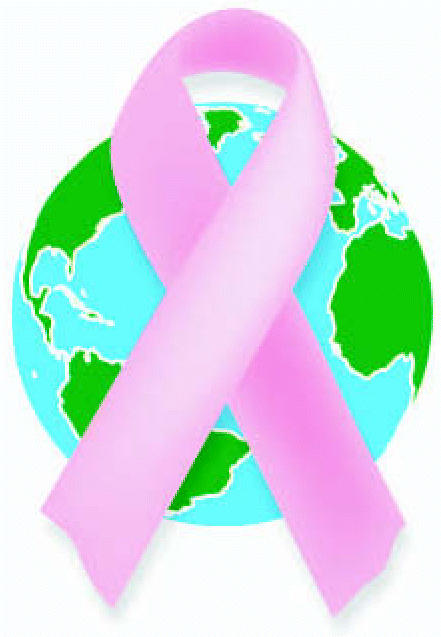


## The End of “Asthma”?

The term “asthma,” probably first used medically by Hippocrates, comes from the Greek for “panting.” Now an editorial in the 16 August 2006 issue of *The Lancet* has called for the scientific community to abandon this term, stating that asthma is not a single disease, but a group of syndromes with different causes and characteristics. The editorial suggests that asthma may actually be only a symptom of several distinct diseases, similar to fever. Currently, an estimated 300 million people in the world have asthma symptoms, and 100 million more are expected to suffer from the condition by the year 2025.

## Got Kenaf?

The market for recyclable materials in Europe is growing, with this year seeing the enaction of a law requiring that all new cars be 85% recyclable. One project that looks to capitalize on this trend is a new manufacturing complex in Spain launched by the UK company SPDG, with $2.51 million provided by the Spanish government and another possible $1.65 million coming from regional governments. The complex, with construction set to begin this year, will manufacture products based on the towering, hibiscus-like plant kenaf. The complex should be able to process 10,000 metric tons of locally grown kenaf each year into recyclable items to replace glass-reinforced plastics and fiberglass in construction, automobiles, and electronics.

**Figure:**
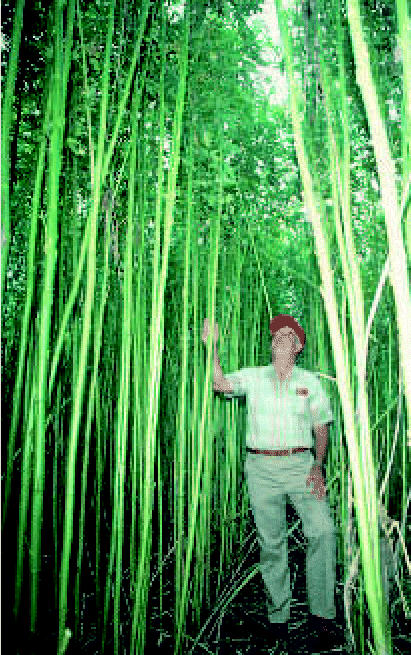


## EU Rules Tough on Toys

The European Union’s Restriction of Hazardous Substances directive, which took effect 1 July 2006, bans lead, mercury, cadmium, hexavalent chromium, polybrominated biphenyls, and polybrominated diphenyl ethers in a wide range of electrical and electronic products—and also bans products that are found to contain these materials. One group that is being hit especially hard by this new policy is Chinese toy makers. In 2005, China exported $15.18 billion worth of toys, including nearly 80% of the toys imported by Europe. Industry experts say the directive will drive manufacturing costs up by at least 20%. The cost of alternative raw materials, now high in demand, is one factor; another is the cost of compliance certification.

**Figure:**
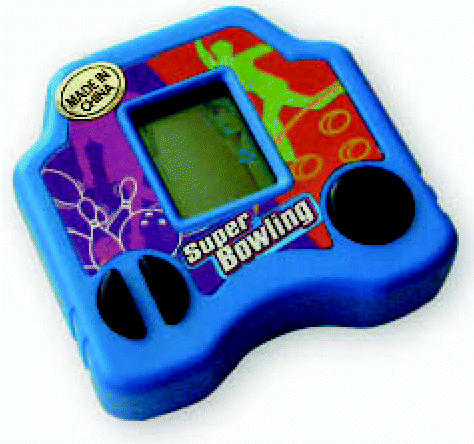


## Clean Air Institute Takes Flight in Latin America

Latin America has 133 cities with populations of more than 500,000. In these cities, transportation is the leading cause of air pollution, which is linked with significant health impacts. In Santiago, Chile, alone, 4,000 premature deaths are linked to air pollution each year. In July 2006, the World Bank announced the creation of the nonprofit Clean Air Institute to manage the Clean Air Initiative for Latin American Cities. This initiative is a coalition of cities, private entities, and NGOs joining together to exchange information on air quality programs. The institute’s main responsibilities include acting as a forum for strategy and project development, and as a center for training and technical assistance.

## Overweight People Now Outnumber the Hungry

With the WHO characterizing obesity as one of the greatest public health challenges of the 21st century, it was announced at the August 2006 meeting of the International Association of Agricultural Economists that the number of overweight people in the world has surpassed the number of malnourished for the first time. Current estimates place the number of overweight or obese people at 1 billion and the number of people without enough to eat at about 800 million, says nutritionist Barry Popkin of the University of North Carolina at Chapel Hill. Though the number of hungry people is falling gradually, the number of obese people is growing rapidly. Popkin suggested that governments should subsidize the production of fruits and vegetables and enact higher taxes on sugary items to help stem the rise in obesity.

**Figure:**